# Bottlenose Dolphin (*Tursiops truncatus*) Metabolic Rate in Relation to Reduced Energy Acquisition

**DOI:** 10.1093/icb/icag134

**Published:** 2026-07-29

**Authors:** Dorian S Houser, Angelo J Incitti, Cassondra L Williams, Daniel E Crocker, Randy E Sacco

**Affiliations:** National Marine Mammal Foundation, San Diego, CA 92106, USA; National Marine Mammal Foundation, San Diego, CA 92106, USA; National Marine Mammal Foundation, San Diego, CA 92106, USA; Sonoma State University, Department of Biology, Rohnert Park, CA 94928, USA; National Animal Disease Center, US Department of Agriculture, Agricultural Research Service, Ames, IA 50010, USA

## Abstract

Interruptions in foraging behavior, displacement from foraging habitats, and reductions in food abundance or quality are common disruptions to energy acquisition experienced by wild animals that may be caused by naturally occurring phenomena or anthropogenic activity. The impact of reduced energy acquisition to an organism is a function of the duration and magnitude of the energy reduction and the physiological response that mitigates the loss of endogenous nutrient reserves. Although the response to reduced energy acquisition has been studied in many terrestrial mammals, little information exists on the response in cetaceans, such as dolphins, which experience metabolic challenges characteristic of a fully aquatic existence. To assess the response of bottlenose dolphins (*Tursiops truncatus*) to dietary energy restriction, five adult dolphins (three male/two female) were subjected to 7 days of a normal diet (ND) followed by 7 days of an energy-restricted diet (ERD) containing 25% less energy per day than the ND. The resting metabolic rate (RMR) was determined for each animal on days three, five, and seven of the ND and ERD periods. Blood samples were collected following each RMR measurement and processed for cortisol, free triiodothyronine (fT3), dehydroepiandrosterone, adiponectin, tumor necrosis factor-α, and interleukin-6, as these hormones and cytokines are known to affect RMR, energy substrate selection, and possibly short-term catabolic signaling or metabolic adaptation. The ERD was associated with a lower RMR, which declined by an average of 5.3% from the ND treatment. The ERD was also associated with lower fT3 levels and increased adiponectin levels, and both showed continuing changes as a function of time on the ERD. Bottlenose dolphins show patterns in RMR and levels of fT3 and adiponectin under dietary energy restriction that are similar to patterns observed in terrestrial mammals. These likely help to conserve endogenous energy stores, increase reliance on lipid oxidation, and protect protein stores. The quantitative information from this study should be useful in modeling the impacts to individuals and populations that experience both natural and human-caused reductions in energy acquisition (e.g., disruptions of foraging, reduced prey quality).

## Introduction

All animals can experience food shortages or changes in food quality. These may occur due to natural phenomena (e.g., drought, climate change) or anthropogenic causes (e.g., habitat degradation, displacement of animals off natural foraging grounds, competition for fish stocks), and can place an animal in a negative energy state that potentially affects both its health and fitness. Among mammals, sufficiently large deficits in energy acquisition trigger physiological responses that conserve overall energy stores and shift substrate use toward greater lipid oxidation and lower protein oxidation until adequate feeding resumes (Castellini and Rea 1992; Wang et al. 2006; McCue 2010). These responses are intermediate to those observed during starvation or the natural fasts experienced by some species, when all energetic costs must be met through the catabolism of endogenous energy reserves. Moderate to severe reductions in energy acquisition over days to weeks produce physiological responses such as reductions in the resting metabolic rate (RMR) (e.g., Forsum et al. 1981; Gonzales-Pacheco et al. 1993; Leibel et al. 1995; Dulloo and Jacquet 1998; Müller et al. 2015; Brinkmann et al. 2016), shifts in the relative levels of cortisol and/or circulating thyroid hormones (Richmond et al. 2010; Wilsterman et al. 2015; Jesmer et al. 2017; Rimbach et al. 2017), and increased rates of lipolysis and levels of circulating fatty acids (Lochmiller et al. 1985; Sticker et al. 1995; Parmley et al. 1996), among others.

Marine mammals encounter the same challenges with prey availability or quality as their terrestrial counterparts, while meeting the additional energy demands of a fully aquatic existence, e.g., maintaining thermoneutrality in water (which transfers heat ~25x faster than air) or managing oxygen stores while diving (e.g., Castellini et al. 1985; Lavigne et al. 1986; Whittow 1987; Kooyman 1989; Williams et al. 1991; Boily 1995; Williams et al. 1996; Yeates and Houser 2008; Meir et al. 2013). These challenges may also overlap with seasonal variations in metabolic rate and associated regulatory hormones (e.g., Kumagai et al. 2006; Rosen and Kumagai 2008; Richmond et al. 2010; Flower et al. 2015; Suzuki et al. 2018, 2024; Houser et al. 2021a; Sherman et al. 2021). Furthermore, there are broad differences in the life histories of many marine mammal species that can affect when impacts to foraging or prey quality are most important. Capital breeding marine mammals segregate periods of foraging from breeding and pupping/calving such that the impact from reductions in energy acquisition are seasonally constrained and reflected in the endogenous energy reserves available for breeding, offspring investment, and maintenance metabolism during natural fasts (Jönsson 1997; Crocker et al. 2001; Costa and Maresh 2022; Christiansen et al. 2025). On the other hand, income breeders are subject to potential disruptions to feeding throughout the year and likely do not show the extreme physiological adaptations to food deprivation observed in capital breeders during their natural fasts (Jönsson 1997; Stephens et al. 2009). Rather, they likely demonstrate an intermediate physiological response to reductions in prey availability or quality that conserves endogenous energy reserves until adequate prey is once again available (e.g., downregulation of the metabolic rate).

Bottlenose dolphins (*Tursiops truncatus*) are income breeders. They are not fasting-adapted and they tend to feed intermittently throughout the day in the wild, spending from 10 to 61% of their activity budget on foraging, depending on the population (Hanson and Defran 1993; Lusseau 2003; Wells et al. 2013; Kassamali-Fox et al. 2020). When deprived of food, bottlenose dolphins quickly transition toward stage II of fasting, wherein lipid oxidation is increased to meet metabolic costs and protein oxidation is minimized to conserve endogenous protein stores (Ortiz et al. 2010; Houser et al. 2021b). However, unless they become ill and incapable of foraging, complete food deprivation is likely an uncommon experience for bottlenose dolphins because of the diversity of prey they consume (Barros and Wells 1998; Pate and McFee 2012; Dunshea et al. 2013; Wells et al. 2013). Reductions in prey availability (e.g., displacement of prey), reductions in prey quality, and disruptions to foraging activity (e.g., through exposure to high-level anthropogenic noise) are more likely to be encountered. These scenarios can place individual dolphins at an energy deficit that could potentially impact health and fitness, if sufficiently persistent. Unfortunately, little information exists on how the bottlenose dolphin physiologically responds to reductions in energy acquisition.

Consistent with what has been observed in other mammals, we hypothesized that the RMR and hormones that affect the RMR, preferential substrate oxidation (e.g., lipid, protein, and carbohydrate), and immune function, would change in response to moderate energy restriction. Specifically, we hypothesized that the RMR and free triiodothyronine (fT3) would decrease under a 7-day period of energy restriction, whereas adiponectin, cortisol, the cortisol to serum dehydroepiandrosterone (DHEA) ratio, tumor necrosis factor-α (TNF-α), and interleukin-6 (IL-6) would increase. Additionally, we hypothesized that if an effect of moderate energy restriction was observed, that changes would continue to occur with the duration that the animal was energy restricted. Free T3 was assessed because it is known to affect the RMR, and cortisol and adiponectin were assessed because they are known to affect fuel substrate selection during dietary energy restriction. The cortisol/DHEA ratio was assessed as a complementary approach to determine whether a stress response was mounted without adequate compensatory or agonistic action of DHEA toward the effects of cortisol (Christeff et al. 1999; Gabai et al. 2020; Whitham et al. 2020; Miller et al. 2021; El-Zawawy et al. 2022). TNF-α and IL-6 were assessed to determine whether the dietary energy restriction was associated with a change in pro-inflammatory cytokines, possibly related to an increased role in short-term catabolic signaling or metabolic adaptation (Wu et al. 2004; Wueest et al. 2014; Montefusco et al. 2021; Aamir et al. 2025). Quantifiable changes in RMR as a function of dietary energy restriction should be useful in modeling potential impacts to bottlenose dolphins experiencing foraging disruptions or reduced prey quality. Similarly, changes in the levels of hormones known to be responsive to energy restriction could provide information on the possible physiological mechanisms used by dolphins to conserve endogenous energy stores under the same conditions.

## Material and methods

Five adult bottlenose dolphins (3M, 2F) ranging in age from 24 to 43 years participated in the study (see Table 1). Neither of the female dolphins were lactating or pregnant at the time of the study and no animals were administered reproductive control medications. The dolphins were part of the U.S. Navy Marine Mammal Program located in San Diego, CA, USA and were housed in netted enclosures within San Diego Bay. Netted enclosures measured either 9 m × 9 m, or 9 m × 18 m, and were connected via underwater gates that could be raised or lowered to control social group mixing. The average water depth at the netted enclosures was ~7 m. The project was approved by the Institutional Animal Care and Use Committee of the Naval Information Warfare Center Pacific and the U.S. Navy Bureau of Medicine and followed all applicable U.S. Department of Defense guidelines for the care and use of animals in research.

**Table 1:** Animal identifier, age, sex, mass at the end of the 7-day ND treatment, and change in mass after the 7-day ERD treatment.

Identifier	Age (yrs)	Sex	Mass (kg; day 7, ND)	Δ Mass (kg)
D1	34	M	257.1	0.0
D2	33	M	195.0	–2.2
D3	24	M	251.7	−4.5
D4	37	F	201.8	−1.3
D5	43	F	186.0	−4.6

### Experimental design

The experiment exposed the dolphins to a normal diet (ND) period and an energy-restricted diet (ERD) period with the ND period occurring immediately prior to the ERD period. All animals participated in both periods, i.e. a repeated measures design, and each period was 1 week long. During the ND period, each dolphin received its ND, as prescribed by its attending veterinarian. The diet consisted of capelin, herring, squid, and threadfin and was fed at quantities to maintain a desired mass. Thus, all animals started at a dietary intake meant to meet maintenance needs while avoiding energy excess or deficits that could bias subsequent measurements. During the ERD period, the quantity of fish was reduced such that the daily energetic intake was 25% less than during the ND period. The RMR was measured and voluntary blood samples were collected on days 3, 5, and 7 of each period. Body mass was measured on the seventh day of each period using either a hanging scale (Brecknell, Fairmont, MN, USA; Model CS; ± 0.3 kg) or a custom platform scale (Arlyn Scales, East Rockaway, NY, USA; ± 0.3 kg). All experimental procedures were performed between January 10 and February 21, 2025 to control for potential seasonal changes in RMR, which have previously been characterized by the authors in this dolphin population (Incitti et al., in press). The back-to-back ND and ERD periods further mitigated any potential for seasonal shifts in RMR within a dolphin during testing. In addition, water temperatures for the region ranged between 13.5 and 16.3°C (NOAA Station SDBC1), which were within the thermal neutral zone of the individual dolphins (Yeates and Houser 2008).

### Oxygen consumption

Rates of oxygen consumption (mL O_2_^.^min^−1^) were measured using open-flow respirometry as previously described (Yeates and Houser 2008). A custom, floating Plexiglass dome (116 cm × 76 cm × 40 cm) was placed and secured in the subject’s netted enclosure. Air was pulled through the dome at a controlled rate from an open incurrent port to a vacuum tube outflow built into the Plexiglass dome. Subject dolphins were trained to rest calmly under the metabolic dome for 16–20 min while maintaining body position using a neoprene-covered PVC biteplate attached to the dome’s frame; i.e. the dolphins did not have to exert effort to stay in position. Each dolphin had more than a year’s worth of experience with the respirometry system prior to the experiment, ensuring that they were desensitized to the procedure and that oxygen consumption rates were unaffected by stress/anxiety due to unfamiliarity with the process.

A mass flow controller (Flowkit 500, Sable Systems International, Henderson, NV, USA) was used to pull air through the metabolic dome at a rate of 250 L^.^min^−1^. Subsamples of excurrent air were collected continuously at a flow rate of 225 mL^.^min^−1^, then passed through desiccant (Drierite, WA Hammond Drierite Company, Xenia, OH, USA) and CO_2_ absorbent (Sodasorb, WR Grace and Company, Cambridge, MA, USA) tubes plumbed in series. Subsamples were then analyzed for oxygen content using a Field Metabolic System (FMS, Sable Systems International) at a sampling rate of 1 Hz. The oxygen content of subsampled air was logged continuously using Expedata software (Sable Systems International) on a laptop computer. In accordance with the manufacturer’s instructions, the O_2_ sensor was calibrated with ambient air and dry nitrogen gas, respectively, and dry nitrogen gas was used to perform weekly leak tests on the metabolic dome. Carbon dioxide was not measured as part of the study.

On days where respirometry sessions occurred, each dolphin received 1.4 kg of fish between 0450 and 0530 h following an overnight fast. Food was then withheld for 3 to 4 h before participating in a respirometry session to ensure the dolphins were postabsorptive (Yeates and Houser 2008). All respirometry sessions were conducted between 0800–1000 h to avoid potential issues associated with diurnal changes in cortisol and thyroid hormones (Houser et al. 2021a). The first 5 min of data from each respirometry session was omitted to account for the animal “settling” into a resting state. Consistent with the criteria for measuring RMR (i.e. Kleiber’s conditions), the dolphins were therefore postabsorptive, mature, nonreproductive, thermally neutral, and resting during oxygen consumption measurements. The remaining data were then analyzed using Expedata’s “Level” tool to identify the most stable 10-min interval, which was used to calculate the mean fractional oxygen content of the excurrent air. Equation (4b) from Withers (1977) was subsequently used to calculate the animal’s RMR as


\begin{eqnarray*}
{{V}_{{{O}_2}}} = \ \frac{{{{V}_{E\ }} \cdot \ \left( {{{F}_{{{I}_{{{O}_2}}}}} - \ F_{{{E}_{{{O}_2}}}}^{\prime}} \right)}}{{1 - \ {{F}_{{{I}_{{{O}_2}}}}} + \ RQ\ \cdot \ \left( {{{F}_{{{I}_{{{O}_2}}}}} - \ F_{{{E}_{{{O}_2}}}}^{\prime}\ } \right)\ }}
\end{eqnarray*}

where ${{V}_{{{O}_2}}}$is the oxygen consumption, ${{V}_E}$ is the rate of excurrent airflow (STDP), ${{F}_{{{I}_{{{O}_2}}}}}$is the fractional concentration of O_2_ for incurrent airflow (STDP), and $F_{{{E}_{{{O}_2}}}}^{\prime}$is the fractional concentration of O_2_ for excurrent airflow (STDP) following the removal of CO_2_. The respiratory quotient (RQ) was assumed to be 0.77 (Williams et al. 2017).

### Blood sampling and processing

Each dolphin voluntarily participated in a blood sample collection immediately following its respirometry session. Dolphins presented the ventral surface of their fluke so that blood could be collected from the arteriovenous plexus with a 21-gauge butterfly blood collection set. Blood collections were made into chilled serum and EDTA blood collection tubes with collection volumes of approximately 10 and 6 mL, respectively. Blood samples were centrifuged at 1090 g for 15 min within 90 min of collection. The serum/plasma supernatant was then stored in a −80°C freezer until analyzed.

Blood samples were processed for cortisol, fT3, DHEA, and adiponectin. Samples were also assessed for TNF-α and IL-6 to assess any potential change in their roles with respect to lipid metabolism and mounting an immune response. Serum cortisol was measured using an antibody-coated tube RIA kit (MP Biomedicals, Irvine, CA; kit #07221102R), previously validated in bottlenose dolphins (Houser et al. 2011, 2021a). Cortisol levels were below detection levels in many samples. To correct for this, the sample volume was increased from 25 to 100 μL and then corrected for the increased sample added, as previously described (Houser et al. 2021a). The average intra-assay coefficient of variation (CV) for cortisol was 1.8%. No interassay CV was determined as all samples were run within a single assay. Both TNF-α and IL-6 were measured using ELISA kits designed for use in the bottlenose dolphin (Kingfisher Biotech, St Paul, MN, USA; kit numbers DIY693P and DIY1417P (Eberle et al. 2018)). Enzyme immunoassays were also used to measure fT3 (AFG Bioscience, Northbrook, IL; kit #EK760296), DHEA (Enzo Life Sciences, Farmingdale, NY; kit# ADI-900-093), and adiponectin (Cayman Chemicals, Ann Arbor, MI; kit# 500641). The assays for fT3, DHEA, and adiponectin were validated for use in the bottlenose dolphin prior to sample processing (parallelism *r*^2^ = 0.98, 0.97, and 0.98, respectively; spike recovery = 103 ± 4%, 105 ± 3%, and 102 ± 4%, respectively). Average intra-assay CVs for fT3, DHEA, and adiponectin were 4.4%, 4.8%, and 4.8%, respectively. As with cortisol, no interassay CV was determined as all samples were run within a single assay.

### Statistical analysis

All statistical tests were performed with JMP (v. 19.0.5; JMP Statistical Discovery LLC, Cary, NC, USA). Data were initially screened for outliers using interquartile plots and an outlier definition of values exceeding 1.5 times the interquartile range. Using this method, a single data point for cortisol was identified as an outlier. The data point was removed prior to further analysis.

Linear mixed models (LMM) with diet (ND or ERD) as a fixed effect and subject as a random effect were run with RMR and each of the hormones and cytokines as the response variable. (Note that IL-6 was not detectable in four of the subjects (Table S1), so no statistical models were run with it as a response variable and it is not discussed further.) Variance components were estimated using restricted maximum likelihood methods. Error distributions were assumed to be Gaussian, and day of sampling was considered a repeated measure. Since the within-animal effect of diet was primarily assessed with the repeated measures design, age, sex, and mass were not explicitly accounted for to avoid over-parameterization and issues with uneven sampling (i.e. mass was only measured twice versus the six measurements for each of the other variables). These stable between-subject differences, which were not modeled as fixed effects, were captured in the subject-specific random effect. However, given the influence of mass on RMR, a variant of the LMM was created in which mass and diet were included as main effects. This required that the single mass measurement for each treatment (ND and ERD) be associated with the three RMR and hormone/cytokine measurements made within each treatment. If diet type was found to be a significant main effect in the initial models, a subsequent LMM was created to assess if changes while under the ERD were affected by the duration spent on the ERD. To this end, sample day (day 3, 5, or 7) within the ERD was used as a fixed effect and subject was preserved as a random effect. If the fixed effect (sample day) was significant, a post hoc comparison (Tukey’s HSD, α = 0.05) was performed to determine which sample days were different from one another.

Model residuals were visually assessed for normality using conditional residual quantile plots, while homoscedasticity was visually assessed using residual by predicted plots. All conditional residual quantile plots demonstrated approximate normality with no more than modest deviations from the expected distribution. Similarly, no evidence of heteroscedasticity or bias was determined from inspection of the residual by predicted plots. No data transforms were therefore required.

## Results

The amount of mass lost by each animal between the end of the ND period and the end of the ERD period is provided in Table 1 (Δ mass). In one of the five dolphins, the amount of mass lost was within the accuracy of the scale used for the measurements (± 0.3 kg). The greatest amount of mass loss was in D5, who lost ~2.5% of her mass over the 7-day ERD. Table S1 (Supplemental Material) presents the RMR and hormone concentrations for each subject by dietary treatment and days within each treatment that samples were collected.

Model results for each of the LMMs assessing the relationship between the response variables (RMR, fT3, cortisol, adiponectin, cortisol/DHEA, and TNF-α) and the fixed effect, diet (ND/ERD), are presented in Table S2 (Supplemental Material). Model results for each of the LMMs assessing the relationship between the response variables (RMR, fT3, and adiponectin) and the fixed effect of sample day under the ERD are presented in Table S3 (Supplemental Material).

**Fig. 1:**
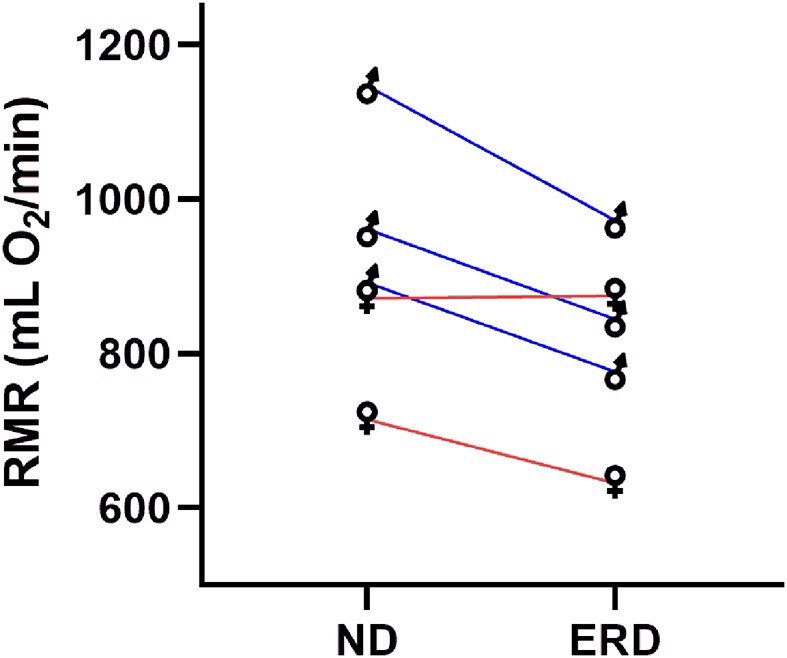
Change in mean RMR of each subject as a function of diet (normal diet = ND, energy-restricted diet = ERD). Symbols indicate the sex of each animal..

Dolphin RMR was higher when dolphins were on the ND than when on the ERD (*β* = 48.6 mL O₂/min, *F*_1, 24.0_ = 4.33, *p* = 0.048; Fig. 1 and Table S2). Using a mean RMR of 917 mL O₂/min while on the ND, this corresponded to an average 5.3% decrease in RMR while dolphins were on the ERD. No effect of the day of sampling while on the ERD was observed (*F*_2, 8_ = 0.98, *p* = 0.417). Including mass in the LMM resulted in only a modest change in the effect of diet (mass: *β* = 3.3 mL O₂/min, *F*_1, 3.0_ = 5.09, *p* = 0.11; diet: *β* = 44.5 mL O₂/min, *F*_1, 24.3_ = 3.62, *p* = 0.069), with the average decrease in RMR while on the ERD reduced to 4.8%. The observed effect of diet was therefore minimally affected by body mass.

**Fig. 2:**
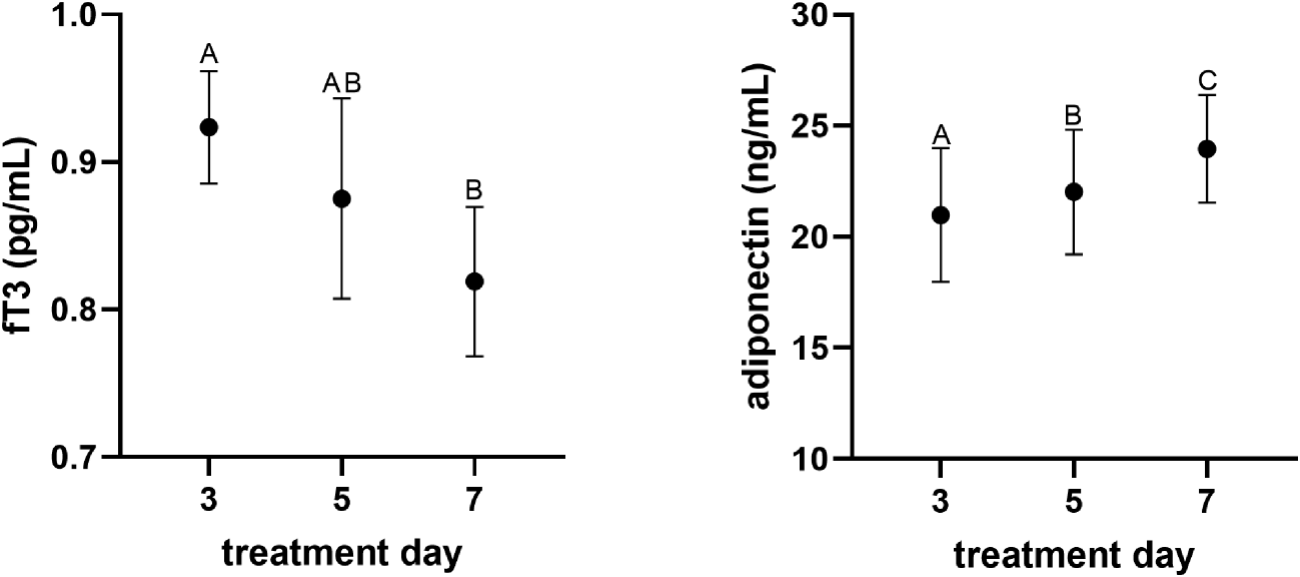
Change in mean (± std dev) levels of (left) fT3 and (right) adiponectin as a function of time on the ERD. Different letters correspond to significant differences between treatment days as determined from a Tukey’s HSD post hoc comparison (*α* = 0.05).

Levels of fT3 were higher when dolphins were on the ND than when on the ERD (*β* = 0.08 pg/mL, *F*_1, 24.0_ = 45.5, *p* < 0.001; Table S2). A significant effect of the day of sampling on fT3 levels while on the ERD was found (*F*_2, 8_ = 9.0, *p* = 0.009; Table S3); effect estimates and *p*-values suggested a decline in fT3 levels, although a post hoc comparison indicated that only the seventh day of the treatment was significantly different from the third day (Tukey’s HSD, α = 0.05; Fig. 2). In contrast to fT3, adiponectin levels were lower when dolphins were on the ND than on the ERD (*β* = −1.42 ng/mL, *F*_1, 24.0_ = 43.2, *p* < 0.0001; Table S2). A significant effect of the day of sampling on adiponectin levels while on the ERD was found (*F*_2, 8_ = 40.8, *p* < 0.0001; Table S3), and effect estimates and *p*-values suggested increasing adiponectin levels with time spent on the ERD. Post hoc analysis suggested all sample days were different from one another (Tukey’s HSD, α = 0.05; Fig. 2). No effect of diet was observed on cortisol (*F*_1, 23.0_ = 1.72, *p* = 0.20), cortisol/DHEA (*F*_1, 23.0_ = 0.43, *p* = 0.52), or TNF-α levels (*F*_1, 24.0_ = 1.39, *p* = 0.25; additional model results for all are presented in Table S2).

## Discussion

Reductions in RMR as a function of reduced energy acquisition protects endogenous nutrient reserves when food is scarce or of poor quality (e.g., low energy content). Though a well-known and observed response among mammals, this phenomenon has been little studied in dolphin species leaving the question as to whether the transition to a fully aquatic existence has affected the adaptive changes in metabolic rate inherited from the dolphin’s terrestrial ancestors. In the study described here, a 25% reduction in energy acquisition was associated with an average reduction in the dolphins’ RMR of 5.3% (or 4.8% if mass is included as a fixed effect in the LMM). Similar patterns have been observed in other vertebrates; i.e. the magnitude of the metabolic rate reduction does not scale equally with reductions in energy acquisition. For example, rats (*Rattus norvegicus*) fed a diet equal to 60% of a baseline diet over a 6-week period reduced basal metabolic rate by 12% (Gonzales-Pacheco et al. 1993); Chinese bulbuls (*Pycnonotus sinensis*) fed a 50% reduced energy diet over 12 days reduced RMR by 10–12% (Zhang et al. 2018); and rhesus monkeys (*Macaca mulatta*) fed a diet 60% of baseline energy content over a 4-month period reduced their total daily energy expenditure, which includes the RMR and additional expenditures (e.g., exercise), by ~24% (Lane et al. 1996). The similarity of metabolic rate reductions between other terrestrial vertebrates and the dolphins suggests that the dolphin has conserved the ancestral response to food shortages, despite the additional energetic challenges (e.g., heat loss) of a fully aquatic existence.

The suggested decline in fT3 as time on the ERD increased is consistent with the role of fT3 in regulating metabolism. Reductions in energy acquisition yielding lower levels of thyroid axis activity, particularly fT3 and tT3, have been observed across many vertebrate species, including marine mammals (e.g., Roth et al. 2002; Rosen and Kumagai 2008; Jeanniard du Dot et al. 2009; Redman et al. 2018). Conversely, adiponectin levels increased with time spent on the ERD. Adiponectin is a protein secreted by adipocytes that increases during periods of dietary energy restriction where loss of adipose tissue occurs (reviewed in Nguyen 2020). Adiponectin promotes the transport and oxidation of fatty acids and the storage of triglycerides in adipose tissue, while also limiting hepatic glucose production (reviewed in Hasenour et al. 2013). It has also been shown to reduce thermogenesis, and thus energy expenditure, in mice (Qiao et al. 2014). Given the complementary nature of fT3 and adiponectin activity, the two hormones likely synergize to facilitate the physiological response to reduced energy acquisition.

Time-dependent changes in fT3 and adiponectin while on the ERD suggest that the dolphins were still dynamically adapting to the energy restriction after 7 days. In dolphins, we know little about the time course over which physiological changes associated with reduced energy acquisition occur, how it might be affected by the magnitude of the dietary energy restriction (e.g., Mitchell et al. 2017), or whether new steady states can be achieved that support survival under a fixed dietary energy restriction. However, for insight, studies can be referenced where the physiological response of dolphins to complete food abstinence (fasting) was assessed. Only a few such studies have been performed. Ortiz et al. (2010) fasted two male dolphins for 38 h and measured changes in serum biochemistry at 14, 24, and 38 h. They found that the fasted bottlenose dolphin behaved in many ways like other (nonfasting adapted) fasting mammals; by 38 h of fasting, nonesterified fatty acids increased by 2.5-fold and blood urea nitrogen decreased by 22%, both physiological responses reflecting protein conservation through increased lipid oxidation and reduced protein oxidation. Houser et al. (2021b) submitted a group of eight dolphins to a 24-h fast and monitored changes in the metabolome between the postabsorptive and 24-h fasted state. As with Ortiz et al. (2010), increases in nonesterified fatty acids and lipid metabolites, and reductions in circulating amino acids and their metabolites, showed a shift toward increased lipid oxidation and protein conservation. A dynamic network marker was identified within the changing metabolome that indicated an emerging negative energy state. Both Ortiz et al. (2010) and Houser et al. (2021b) concluded that when deprived of food dolphins rapidly transition toward a physiological state like stage II of fasting, which is characterized by a reduction in the metabolic rate, a shift toward lipid oxidation to meet metabolic costs, and a shift away from protein oxidation to conserve endogenous protein stores. The results of the current study, both with respect to the dietary treatment effect on RMR and time-dependent changes in fT3 and adiponectin while on the ERD, are consistent with these predictions. However, the response is muted, likely intermediate to that of the fasting response, and may continue to gradually change as the energy restriction duration increases.

An important limitation to the current study is that it characterizes the response to dietary energy restriction at a specific time of year (winter). Many animals show seasonal changes in the RMR and thyroid hormones that are potentially modulated by food availability, photoperiod, or temperature, and which may be accompanied by reductions in activity level (Silver et al. 1969; Weiner 1977; Moen 1978; Blaxter and Boyne 1982; Baldock et al. 1988; Dahl et al. 1994; Timisjärvi et al. 1994; Dahl et al. 1995; Worden and Pekins 1995; Thrun et al. 1997; Yokus et al. 2006; Turbill et al. 2011; Riek et al. 2017; Rimbach et al. 2017; Webster et al. 1991a , 1991b). Recently, seasonal/photoperiod variations in the RMR and thyroid hormone profiles have been demonstrated in the bottlenose dolphin (Incitti et al., in press; Houser et al. 2021a). It seems reasonable that a changing baseline RMR, and baseline changes in the hormones that regulate the RMR, could alter the degree of RMR suppression and the time course over which it occurs. This limitation could be addressed by repeating the study in different seasons. Similarly, since the magnitude of the energy restriction also affects the RMR, repeating the study with an increased energy restriction (e.g., 50% energy reduction) would provide additional data to characterize the dynamics of the metabolic response as a function of the degree of dietary energy restriction. It should be noted that animal training staff did not note any change in the activity level of the dolphins while on the ERD, nor have changes in activity level been noted with seasonal/photoperiod-related changes in RMR, suggesting that the changes in RMR are not activity dependent.

Energy expenditure in relation to energy acquisition has lacked quantitative characterization in marine mammals, yet this relationship is critical to predicting potential impacts of human activity that disrupt foraging behavior, displace prey, or displace marine mammals from their preferred foraging sites (McHuron et al. 2022; Noren and Rosen 2023). Reductions in prey availability or quality as a function of either natural or anthropogenic events are a likely stressor experienced by wild marine mammals. Naturally occurring changes, e.g. El Niño Southern Oscillation, can produce changes in prey availability/quality that range in impact from negligible to greatest concern for marine mammal populations and stocks (Urbán R. et al. 2003; Le Boeuf and Crocker 2005; DeRango et al. 2019). Similarly, anthropogenic activities may cause long-term habitat disruption, displacement from foraging habitats, or displacement of prey (e.g., Pirotta et al. 2013), or may only cause temporary displacements from foraging sites or of prey species (e.g., McCarthy et al. 2011). Recent decades have seen the development of models that seek to predict the outcome of stressors on marine mammals, including the impact of reduced energy acquisition (reviewed in Pirotta et al. 2022). Unfortunately, these models commonly lack quantitative data on the impact of a stressor to an individual that can then be used to estimate impacts at the population level. This study has provided quantitative data on changes in the RMR that occur as a function of reduced energy acquisition in the bottlenose dolphin. The data are directly applicable to predictive impact models and additional exploration of the how suppression of the RMR is affected by the duration and magnitude of energy restriction should help modelers better predict the ability of marine mammals to tolerate or be impacted by foraging disruptions or reduced prey quality.

## Supplementary Material

icag134_Supplemental_File

## Data Availability

The data underlying this article are included in the Supplemental Material.

## Notes

From the symposium “Hormones at sea: New frontiers in marine mammal endocrinology and ecophysiology” presented at the annual meeting of the Society for Integrative and Comparative Biology, January 3–7th, 2026.

## References

[bib1] Aamir AB, Kumari R, Latif R, Ahmad S, Rafique N, Salem AM, Alasoom LI, Alsunni A, Alabdulhadi AS, Chander S et al. 2025. Effects of intermittent fasting and caloric restriction on inflammatory biomarkers in individuals with obesity/overweight: a systematic review and meta-analysis of randomized controlled trials. Obesity Rev. 26:e13838. 10.1111/obr.13838. 39289905

[bib2] Baldock NM, Sibly RM, Penning PD. 1988. Behaviour and seasonal variation in heart rate in domestic sheep, *Ovis aries*. Anim Behav. 36:35–43. 10.1016/S0003-3472(88)80247-1.

[bib3] Barros NB, Wells RS. 1998. Prey and feeding patterns of resident bottlenose dolphins (*Tursiops truncatus*) in Sarasota Bay, Florida. J Mammal. 79:1045–59. 10.2307/1383114.

[bib4] Blaxter KL, Boyne AW. 1982. Fasting and maintenance metabolism of sheep. J Agric Sci. 99:611–20. 10.1017/S0021859600031294.

[bib5] Boily P . 1995. Theoretical heat flux in water and habitat selection of phocid seals and beluga whales during the annual molt. J Theor Biol. 172:235–44. 10.1006/jtbi.1995.0020.

[bib6] Brinkmann L, Gerken M, Hambly C, Speakman JR, Riek A. 2016. Thyroid hormones correlate with field metabolic rate in ponies, *Equus ferus caballus*. J Exp Biol. 219:2559–66. 27312472 10.1242/jeb.138784

[bib7] Castellini MA, Murphy BJ, Fedak MA, Ronald K, Gofton N, Hochachka PW. 1985. Potentially conflicting metabolic demands of diving and exercise in seals. J Appl Physiol. 58:392–9. 10.1152/jappl.1985.58.2.392. 3980347

[bib8] Castellini MA, Rea LD. 1992. The biochemistry of natural fasting at its limits. Experientia. 48:575–82. 10.1007/BF01920242. 1612138

[bib9] Christeff N, Melchior JC, Mammes O, Gherbi N, Dalle MT, Nunez EA. 1999. Correlation between increased cortisol:DHEA ratio and malnutrition in HIV-positive men. Nutrition. 15:534–9. 10.1016/S0899-9007(99)00111-2. 10422082

[bib10] Christiansen F, Madsen PT, Andrews-Goff V, Double MC, How JR, Clapham P, Ivashchenko Y, Tormosov D, Sprogis KR. 2025. Extreme capital breeding for giants: effects of body size on humpback whale energy expenditure and fasting endurance. Ecol Modell. 501:110994. 10.1016/j.ecolmodel.2024.110994.

[bib11] Costa DP, Maresh JL. 2022. Reproductive energetics of phocids. In: Costa DP, McHuron EA, editors. Ethology and behavioral ecology of phocids. Switzerland: Springer International Publishing 281–309.

[bib12] Crocker DE, Williams JD, Costa DP, Le Boeuf BJ. 2001. Maternal traits and reproductive effort in northern elephant seals. Ecology. 82:3541–55. 10.1890/0012-9658(2001)082[3541:MTAREI]2.0.CO;2.

[bib13] Dahl GE, Evans NP, Moenter SM, Karsch FJ. 1994. The thyroid gland is required for reproductive neuroendocrine responses to photoperiod in the ewe. Endocrinology. 135:10–5. 10.1210/endo.135.1.8013340. 8013340

[bib14] Dahl GE, Evans NP, Thrun LA, Karsch FJ. 1995. Thyroxine is permissive to seasonal transitions in reproductive neuroendocrine activity in the ewe. Biol Reprod. 52:690–6. 10.1095/biolreprod52.3.690. 7756463

[bib15] DeRango EJ, Prager KC, Greig DJ, Hooper AW, Crocker DE. 2019. Climate variability and life history impact stress, thyroid, and immune markers in California sea lions (*Zalophus californianus*) during El Niño conditions. Conserv Physiol. 7. 10.1093/conphys/coz010. PMC6518924 31110762

[bib16] Dulloo AG, Jacquet J. 1998. Adaptive reduction in basal metabolic rate in response to food deprivation in humans: a role for feedback signals from fat stores. Am J Clin Nutr. 68:599–606. 10.1093/ajcn/68.3.599. 9734736

[bib17] Dunshea G, Barros NB, Berens McCabe EJ, Gales NJ, Hindell MA, Jarman SN, Wells RS. 2013. Stranded dolphin stomach contents represent the free-ranging population’s diet. Biol Lett. 9. 10.1098/rsbl.2012.1036. PMC3645016 23637389

[bib18] Eberle KC, Venn-Watson S, Jensen ED, LaBresh J, Sullivan Y, Kakach L, Sacco RE. 2018. Development and testing of species-specific ELISA assays to measure IFN-γ and TNF-α in bottlenose dolphins (*Tursiops truncatus*). PLoS One. 13:e0190786. 29304133 10.1371/journal.pone.0190786 PMC5755893

[bib19] El-Zawawy HT, El-Aghoury AA, Katri KM, El-Sharkawy EM, Gad SMS. 2022. Cortisol/DHEA ratio in morbidly obese patients before and after bariatric surgery: relation to metabolic parameters and cardiovascular performance. Int J Obes. 46:381–92. 10.1038/s41366-021-00997-x. 34725442

[bib20] Flower JE, Allender MC, Givanelli RP, Summers SD, Spoon TR, St. Ledger JA, Goertz CE, Dunn L, Romano TA, Hobbs RC et al. 2015. Circulating concentrations of thyroid hormone in beluga whales (*Delphinapterus leucas*): influence of age, sex, and season. J Zoo Wildl Med. 46:456–67. 10.1638/2014-0127.1. 26352948

[bib21] Forsum E, Hillman PE, Nesheim MC. 1981. Effect of energy restriction on total heat production, basal metabolic rate, and specific dynamic action of food in rats. J Nutr. 111:1691–7. 10.1093/jn/111.10.1691. 6793699

[bib22] Gabai G, Mongillo P, Giaretta E, Marinelli L. 2020. Do dehydroepiandrosterone (DHEA) and its sulfate (DHEAS) play a role in the stress response in domestic animals?. Front Vet Sci. 7:588835. 10.3389/fvets.2020.588835. 33195624 PMC7649144

[bib23] Gonzales-Pacheco DM, Buss WC, Koehler KM, Woodside WF, Alpert SS. 1993. Energy restriction reduces metabolic rate in adult male Fisher-344 rats. J Nutr. 123:90–7. 10.1093/jn/123.1.90. 8421235

[bib24] Hanson MT, Defran RH. 1993. The behaviour and feeding ecology of the Pacific coast bottlenose dolphin, *Tursiops truncatus*. Aquat Mammal. 19:127–42.

[bib25] Hasenour CM, Berglund ED, Wasserman DH. 2013. Emerging role of AMP-activated protein kinase in endocrine control of metabolism in the liver. Mol Cell Endocrinol. 366:152–62. 10.1016/j.mce.2012.06.018. 22796337 PMC3538936

[bib26] Houser DS, Champagne CD, Wasser SK, Booth RK, Romano T, Crocker DE. 2021a. Influence of season, age, sex, and time of day on the endocrine profile of the common bottlenose dolphin (*Tursiops truncatus*). Gen Comp Endocrinol. 313:113889. 10.1016/j.ygcen.2021.113889. 34425086

[bib27] Houser DS, Derous D, Douglas A, Lusseau D. 2021b. Metabolic response of dolphins to short-term fasting reveals physiological changes that differ from the traditional fasting model. J Exp Biol. 224. 10.1242/jeb.238915. PMC8126448 33766933

[bib28] Houser DS, Yeates LC, Crocker DE. 2011. Cold stress induces an adrenocortical response in bottlenose dolphins (*Tursiops truncatus*). J Zoo Wildl Med. 42:565–71. 10.1638/2010-0121.1. 22204049

[bib29] Incitti AJ, Williams CL, Houser DS, Crocker DE. In Press. Seasonal variation in resting metabolic rate, thyroid hormones, and cortisol in the bottlenose dolphin (Tursiops truncatus). J Exp Biol. 10.1242/jeb.251558 42533563

[bib30] Jeanniard du Dot T, Rosen DAS, Richmond JP, Kitaysky AS, Zinn SA, Trites AW. 2009. Changes in glucocorticoids, IGF-1 and thyroid hormones as indicators of nutritional stress and subsequent refeeding in Steller sea lions (*Eumetopias jubatus*). Comparative Biochem Physiol A Mol Integr Physiol. 152:524–34. 10.1016/j.cbpa.2008.12.010. 19146974

[bib31] Jesmer BR, Goheen JR, Monteith KL, Kauffman MJ. 2017. State-dependent behavior alters endocrine–energy relationship: implications for conservation and management. Ecol Appl. 27:2303–12. 10.1002/eap.1608. 28777884

[bib32] Jönsson KI . 1997. Capital and income breeding as alternative tactics of resource use in reproduction. Oikos. 78:57–66. 10.2307/3545800.

[bib33] Kassamali-Fox A, Christiansen F, May-Collado LJ, Ramos EA, Kaplin BA. 2020. Tour boats affect the activity patterns of bottlenose dolphins (*Tursiops truncatus*) in Bocas Del Toro, Panama. Peer J. 8:e8804. 10.7717/peerj.8804. 32266117 PMC7115753

[bib34] Kooyman GL . 1989. Diverse divers: physiology and behaviour. Berlin: Springer-Verlag.

[bib35] Kumagai S, Rosen DAS, Trites AW. 2006. Body mass and composition responses to short-term low energy intake are seasonally dependent in Steller sea lions (*Eumetopias jubatus*). J Comp Physiol B. 176:589–98. 10.1007/s00360-006-0082-y. 16625362

[bib36] Lane MA, Baer DJ, Rumpler WV, Weindruch R, Ingram DK, Tilmont EM, Cutler RG, Roth GS. 1996. Calorie restriction lowers body temperature in Rhesus monkeys, consistent with a postulated anti-aging mechanism in rodents. Proc Natl Acad Sci USA. 93:4159–64. 10.1073/pnas.93.9.4159. 8633033 PMC39504

[bib37] Lavigne DM, Innes S, Worthy GAJ, Kovacs KM, Schmitz OJ, Hickie JP. 1986. Metabolic rates of seals and whales. Can J Zool. 64:279–84. 10.1139/z86-047.

[bib38] Le Boeuf BJ, Crocker DE. 2005. Ocean climate and seal condition. BMC Biol. 3::10.1186/1741-7007-1183-1189. PMC1082885 15794819

[bib39] Leibel RL, Rosenbaum M, Hirsch J. 1995. Changes in energy expenditure resulting from altered body weight. N Engl J Med. 332:621–8. 10.1056/NEJM199503093321001. 7632212

[bib40] Lochmiller RL, Varner LW, Grant WE. 1985. Metabolic and hormonal responses to dietary restriction in adult female collared peccaries. J Wildl Manag. 49:733–41. 10.2307/3801703.

[bib41] Lusseau D . 2003. Effects of tour boats on the behavior of bottlenose dolphins: using Markov chains to model anthropogenic impacts. Conserv Biol. 17:1785–93. 10.1111/j.1523-1739.2003.00054.x.

[bib42] McCarthy E, Moretti D, Thomas L, DiMarzio N, Morrissey R, Jarvis S, Ward J, Izzi A, Dilley A. 2011. Changes in spatial and temporal distribution and vocal behavior of blainville’s beaked whales (*Mesoplodon densirostris*) during multiship exercises with mid-frequency sonar. Mar Mammal Sci. 27:E206–26. 10.1111/j.1748-7692.2010.00457.x.

[bib43] McCue MD . 2010. Starvation physiology: reviewing the different strategies animals use to survive a common challenge. Comp Biochem Physiol Part A Mol Integr Physiol. 156:1–18. 10.1016/j.cbpa.2010.01.002. 20060056

[bib44] McHuron EA, Adamczak S, Arnould JPY, Ashe E, Booth C, Bowen WD, Christiansen F, Chudzinska M, Costa DP, Fahlman A et al. 2022. Key questions in marine mammal bioenergetics. Cons Physiol. 10:coac055. 10.1093/conphys/coac055 PMC9358695 35949259

[bib45] Meir JU, Robinson PW, Vilchis LI, Kooyman GL, Costa DP, Ponganis PJ. 2013. Blood oxygen depletion is independent of dive function in a deep diving vertebrate, the Northern elephant seal. PLoS One. 8:e83248. 10.1371/journal.pone.0083248. 24376671 PMC3871621

[bib46] Miller LJ, Lauderdale LK, Walsh MT, Bryant JL, Mitchell KA, Granger DA, Mellen JD. 2021. Reference intervals and values for fecal cortisol, aldosterone, and the ratio of cortisol to dehydroepiandrosterone metabolites in four species of cetaceans. PLoS One. 16:e0250331. 10.1371/journal.pone.0250331. 34460862 PMC8404979

[bib47] Mitchell SE, Tang Z, Kerbois C, Delville C, Derous D, Green CL, Wang Y, Han JJ, Chen L, Douglas A et al. 2017. The effects of graded levels of calorie restriction: VIII. Impact of short term calorie and protein restriction on basal metabolic rate in the c57bl/6 mouse. Oncotarget. 8:17453–74. 10.18632/oncotarget.15294. 28193912 PMC5392262

[bib48] Moen AN . 1978. Seasonal changes in heart rates, activity, metabolism, and forage intake of white-tailed deer. J Wildl Manag. 42:715–38. 10.2307/3800763.

[bib49] Montefusco L, D’Addio F, Loretelli C, Ben Nasr M, Garziano M, Rossi A, Pastore I, Plebani L, Lunati ME, Bolla AM et al. 2021. Anti-inflammatory effects of diet and caloric restriction in metabolic syndrome. J Endocrinol Invest. 44:2407–15. 10.1007/s40618-021-01547-y. 33686615 PMC8502121

[bib50] Müller MJ, Enderle J, Pourhassan M, Braun W, Eggeling B, Lagerpusch M, Glüer C-C, Kehayias JJ, Kiosz D, Bosy-Westphal A et al. 2015. Metabolic adaptation to caloric restriction and subsequent refeeding: the Minnesota starvation experiment revisited. Am J Clin Nutr. 102:807–19. 26399868 10.3945/ajcn.115.109173

[bib51] Nguyen TMD . 2020. Adiponectin: role in physiology and pathophysiology. Int J Prev Med. 11:136. 10.4103/ijpvm.IJPVM_193_20. 33088464 PMC7554603

[bib52] Noren SR, Rosen DAS. 2023. What are the metabolic rates of marine mammals and what factors impact this value: a review. Cons Physiol. 11:coad077. 10.1093/conphys/coad077 PMC10545007 37790839

[bib53] Ortiz RM, Long B, Casper D, Ortiz CL, Williams TM. 2010. Biochemical and hormonal changes during acute fasting and re-feeding in bottlenose dolphins (*Tursiops truncatus*). Mar Mammal Sci. 26:409–19. 10.1111/j.1748-7692.2009.00309.x.

[bib54] Ortiz RM, Wade CE, Ortiz CL. 2001. Effects of prolonged fasting on plasma cortisol and TH in postweaned northern elephant seal pups. Am J Physiol Regul Integr Comp Physiol. 280:R790–5. 10.1152/ajpregu.2001.280.3.R790. 11171659

[bib55] Parmley KLS, Machado CR, McNamara JP. 1996. Rates of lipid metabolism in adipose tissue of pigs adapt to lactational state and dietary energy restriction. J Nutr. 126:1644–56. 10.1093/jn/126.6.1644. 8648439

[bib56] Pate SM, McFee WE. 2012. Prey species of bottlenose dolphins (*Tursiops truncatus*) from South Carolina waters. Southeast Nat. 11:1–22. 10.1656/058.011.0101.

[bib57] Pirotta E, Laesser BE, Hardaker A, Riddoch N, Marcoux M, Lusseau D. 2013. Dredging displaces bottlenose dolphins from an urbanised foraging patch. Mar Pollut Bull. 74:396–402. 10.1016/j.marpolbul.2013.06.020. 23816305

[bib58] Pirotta E, Thomas L, Costa DP, Hall AJ, Harris CM, Harwood J, Kraus SD, Miller PJ, Moore M, Photopoulou T et al. 2022. Understanding the combined effects of multiple stressors: a new perspective on a longstanding challenge. Sci Total Environ, 821, 153322. 10.1016/j.scitotenv.2022.153322. 35074373

[bib59] Qiao L, Yoo H, Bosco C, Lee B, Feng GS, Schaack J, Chi NW, Shao J. 2014. Adiponectin reduces thermogenesis by inhibiting brown adipose tissue activation in mice. Diabetologia. 57:1027–36. 10.1007/s00125-014-3180-5. 24531262 PMC4077285

[bib60] Redman LM, Smith SR, Burton JH, Martin CK, Il’yasova D, Ravussin E. 2018. Metabolic slowing and reduced oxidative damage with sustained caloric restriction support the rate of living and oxidative damage theories of aging. Cell Metab. 27:805–815.e4. 10.1016/j.cmet.2018.02.019. 29576535 PMC5886711

[bib61] Richmond JP, Jeanniard du Dot T, Rosen DA, Zinn SA. 2010. Seasonal influence on the response of the somatotropic axis to nutrient restriction and re-alimentation in captive Steller sea lions (*Eumetopias jubatus*). J Exp Zool. 313A:144–56. 10.1002/jez.584. 20084663

[bib62] Riek A, Brinkmann L, Gauly M, Perica J, Ruf T, Arnold W, Hambly C, Speakman JR, Gerken M. 2017. Seasonal changes in energy expenditure, body temperature and activity patterns in llamas (*Lama glama*). Sci Rep. 7:7600. 10.1038/s41598-017-07946-7. 28790450 PMC5548813

[bib63] Rimbach R, Pillay N, Schradin C. 2017. Both thyroid hormone levels and resting metabolic rate decrease in african striped mice when food availability decreases. J Exp Biol. 220:837–43. 27994044 10.1242/jeb.151449

[bib64] Rosen DAS, Kumagai S. 2008. Hormone changes indicate that winter is a critical period for food shortages in Steller sea lions. J Comp Physiol B. 178:573–83. 10.1007/s00360-007-0247-3. 18210131

[bib65] Roth GS, Handy AM, Mattison JA, Tilmont EM, Ingram DK, Lane MA. 2002. Effects of dietary caloric restriction and aging on thyroid hormones of rhesus monkeys. Horm Metab Res. 34:378–82. 10.1055/s-2002-33469. 12189585

[bib66] Sherman KK, Beaulieu-McCoy NE, Wurster EL, Wells RS, Smith CR, Barleycorn AA, Allen JB, Kellar NM. 2021. Serum correlation, demographic differentiation, and seasonality of blubber testosterone in common bottlenose dolphins, *Tursiops truncatus*, in Sarasota Bay, FL. Sci Rep. 11:8941. 10.1038/s41598-021-88602-z. 33903714 PMC8076180

[bib67] Silver H, Colovos NF, Holter JB, Hayes HH. 1969. Fasting metabolism of white-tailed deer. J Wildlife Manag. 33:490–8. 10.2307/3799370.

[bib68] Stephens PA, Boyd IL, McNamara JM, Houston AI. 2009. Capital breeding and income breeding: their meaning, measurement, and worth. Ecology. 90:2057–67. 10.1890/08-1369.1. 19739368

[bib69] Sticker LS, Thompson DL Jr., Bunting LD, Fernandez JM, DePew CL. 1995. Dietary protein and(or) energy restriction in mares: plasma glucose, insulin, nonesterified fatty acid, and urea nitrogen responses to feeding, glucose, and epinephrine. J Anim Sci. 73:136–44. 10.2527/1995.731136x. 7601726

[bib70] Suzuki M, Banno K, Usui T, Funasaka N, Segawa T, Kirihata T, Kamisako H, Ueda K, Munakata A. 2018. Seasonal changes in plasma levels of thyroid hormones and the effects of the hormones on cellular ATP content in common bottlenose dolphin. Gen Comp Endocrinol. 262:20–6. 10.1016/j.ygcen.2018.03.008. 29524524

[bib71] Suzuki M, Funasaka N, Sato Y, Inamori D, Watanabe Y, Ozaki M, Hosono M, Shindo H, Kawamura K, Tatsukawa T et al. 2024. Association of seasonal changes in circulating cortisol concentrations with the expression of cortisol biosynthetic enzymes and a glucocorticoid receptor in the blubber of common bottlenose dolphin. Gen Comp Endocrinol. 352:114516. 10.1016/j.ygcen.2024.114516. 38593942

[bib72] Thrun LA, Dahl GE, Evans NP, Karsch FJ. 1997. A critical period for thyroid hormone action on seasonal changes in reproductive neuroendocrine function in the ewe. Endocrinology. 138:3402–9. 10.1210/endo.138.8.5341. 9231794

[bib73] Timisjärvi J, Ojutkangas V, Eloranta E, Nieminen M, Leppäluoto J, Liimatainen S, Vuolteenaho O. 1994. Annual variations in serum thyroid-stimulating hormone and thyroid hormones and in their responses to thyrotrophin-releasing hormone in the reindeer. J Endocrinol. 141:527–33. 10.1677/joe.0.1410527. 8071651

[bib74] Turbill C, Ruf T, Mang T, Arnold W. 2011. Regulation of heart rate and rumen temperature in red deer: effects of season and food intake. J Exp Biol. 214:963–70. 10.1242/jeb.052282. 21346124 PMC3280896

[bib75] Urbán RJ, Gómez-Gallardo A, Ludwig S. 2003. Abundance and mortality of gray whales at Laguna San Ignacio, Mexico, during the 1997-98 El Niño and the 1998-99 La Niña. Geofís Int. 42:439–46.

[bib76] Wang T, Hung CCY, Randall DJ. 2006. The comparative physiology of food deprivation: from feast to famine. Annu Rev Physiol. 68:223–51. 10.1146/annurev.physiol.68.040104.105739. 16460272

[bib77] Webster JR, Moenter SM, Barrell GK, Lehman MN, Karsch FJ. 1991a. Role of the thyroid gland in seasonal reproduction. III. Thyroidectomy blocks seasonal suppression of gonadotropin-releasing hormone secretion in sheep. Endocrinology. 129:1635–43. 10.1210/endo-129-3-1635. 1874193

[bib78] Webster JR, Moenter SM, Woodfill CJI, Karsch FJ. 1991b. Role of the thyroid gland in seasonal reproduction. II. Thyroxine allows a season-specific suppression of gonadotropin secretion in sheep. Endocrinology. 129:176–83. 10.1210/endo-129-1-176. 2055181

[bib79] Weiner J . 1977. Energy metabolism of the roe deer. Acta Theriol. 22:3–24. 10.4098/AT.arch.77-1.

[bib80] Wells RS, McHugh KA, Douglas DC, Shippee S, McCabe EB, Barros NB, Phillips GT. 2013. Evaluation of potential protective factors against metabolic syndrome in bottlenose dolphins: feeding and activity patterns of dolphins in Sarasota Bay, Florida. Front Endocrinol. 4:139. 10.3389/fendo.2013.00139 PMC3794307 24133483

[bib81] Whitham JC, Bryant JL, Miller LJ. 2020. Beyond glucocorticoids: integrating dehydroepiandrosterone (DHEA) into animal welfare research. Animals. 10:1381. 10.3390/ani10081381. 32784884 PMC7459918

[bib82] Whittow GC . 1987. Thermoregulatory adaptations in marine mammals: interacting effects of exercise and body mass. A review. Mar Mammal Sci. 3:220–41. 10.1111/j.1748-7692.1987.tb00165.x.

[bib83] Williams TM, Kendall TL, Richter BP, Ribeiro-French CR, John JS, Odell KL, Losch BA, Feuerbach DA, Stamper MA. 2017. Swimming and diving energetics in dolphins: a stroke-by-stroke analysis for predicting the cost of flight responses in wild odontocetes. J Exp Biol. 220:1135–45. 10.1242/jeb.154245. 28298467

[bib84] Williams TM, Kooyman GL, Croll DA. 1991. The effect of submergence on heart rate and oxygen consumption of swimming seals and sea lions. J Comp Physiol B. 160:637–44. 10.1007/BF00571261. 2045544

[bib85] Williams TM, Shippee SF, Rothe MJ. 1996. Strategies for reducing foraging costs in dolphins. In: Greenstreet S, Tasker ML, editors. Aquatic predators. London: Blackwell Science Ltd. p.4–9.

[bib86] Wilsterman K, Buck CL, Barnes BM, Williams CT. 2015. Energy regulation in context: free-living female arctic ground squirrels modulate the relationship between thyroid hormones and activity among life history stages. Horm Behav. 75:111–9. 10.1016/j.yhbeh.2015.09.003. 26416501

[bib87] Withers PC . 1977. Measurement of VO_2_, VCO_2_, and evaporative water loss with a flow-through mask. J Appl Physiol. 42:120–3. 10.1152/jappl.1977.42.1.120. 833070

[bib88] Worden KA, Pekins PJ. 1995. Seasonal change in feed intake, body composition, and metabolic rate of white-tailed deer. Can J Zool. 73:452–7. 10.1139/z95-052.

[bib89] Wu G, Brouckaert P, Olivecrona T. 2004. Rapid downregulation of adipose tissue lipoprotein lipase activity on food deprivation: evidence that TNF-α is involved. Am J Physiol Endocrinol Metab. 286:E711–7. 10.1152/ajpendo.00257.2003. 14693508

[bib90] Wueest S, Item F, Boyle CN, Jirkof P, Cesarovic N, Ellingsgaard H, Böni-Schnetzler M, Timper K, Arras M, Donath MY et al. 2014. Interleukin-6 contributes to early fasting-induced free fatty acid mobilization in mice. Am J Physiol Regul Integr Comp Physiol. 306:R861–7. 10.1152/ajpregu.00533.2013. 24694381

[bib91] Yeates LC, Houser DS. 2008. Thermal tolerance in bottlenose dolphins (*Tursiops truncatus*). J Exp Biol. 211:3249–57. 10.1242/jeb.020610. 18840658

[bib92] Yokus B, Cakir DU, Kanay Z, Gulten T, Uysal E. 2006. Effects of seasonal and physiological variations on the serum chemistry, vitamins and thyroid hormone concentrations in sheep. J Vet Med Ser A. 53:271–6. 10.1111/j.1439-0442.2006.00831.x. 16901267

[bib93] Zhang Y, Yang K, Yang P, Su Y, Zheng W, Liu J. 2018. Food restriction decreases BMR, body and organ mass, and cellular energetics, in the Chinese bulbul (*Pycnonotus sinensis*). Avian Res. 9:39. 10.1186/s40657-018-0131-8.

